# A Systematic Methodology to Analyze the Impact of Hand-Rim Wheelchair Propulsion on the Upper Limb

**DOI:** 10.3390/s19214643

**Published:** 2019-10-25

**Authors:** Blanca Larraga-García, Vicente Lozano-Berrio, Álvaro Gutiérrez, Ángel Gil-Agudo, Antonio J. del-Ama

**Affiliations:** 1 Escuela Técnica Superior de Ingenieros de Telecomunicación, Universidad Politéncia de Madrid, Av. Complutense 30, 28040 Madrid, Spain; a.gutierrez@upm.es; 2 Biomechanics and Technical Aids Unit, National Hospital for Paraplegics, Finca La Peraleda, 45071 Toledo, Spain; vlozanob@sescam.jccm.es (V.L.-B.); amgila@sescam.jccm.es (Á.G.-A.); antonio.delama@urjc.es (A.J.d.-A.); 3 Rey Juan Carlos University, Calle Tulipán, 0, 28933 Móstoles, Madrid, Spain; ajdela@sescam.jccm.es

**Keywords:** methodology, spinal cord injury, biomechanics, wheelchairs, upper limb

## Abstract

Manual wheelchair propulsion results in physical demand of the upper limb extremities that, because of its repetitive nature, can lead to chronic pathologies on spinal cord injury patients. The aim of this study was to design and test a methodology to compare kinematic and kinetic variables of the upper limb joints when propelling different wheelchairs. Moreover, this methodology was used to analyze the differences that may exist between paraplegic and tetraplegic patients when propelling two different wheelchairs. Five adults with paraplegia and five adults with tetraplegia performed several propulsion tests. Participants propelled two different wheelchairs for three minutes at 0.833 m/s (3 km/h) with one minute break between the tests. Kinematic and kinetic variables of the upper limb as well as variables with respect to the propulsion style were recorded. Important differences in the kinetic and kinematic variables of the joints of the upper limb were found when comparing paraplegic and tetraplegic patients. Nevertheless, this difference depends on the wheelchair used. As expected, in all tests, the shoulder shows to be the most impacted joint.

## 1. Introduction

Life expectancy of people who suffer from a spinal cord injury has increased during the last decades thanks to the development of specific treatments, technology evolution, medical care and rehabilitation techniques. This has led to research on the chronic conditions of patients with spinal cord injury [1]. For most of these patients, the wheelchair is their way to move independently. Therefore, it is important to study the consequences of long-term wheelchair use, particularly manual wheelchair use. Pain, impairments and injuries in the upper limb may have a strong impact in these patients conditioning their quality of living [2]. However, for people with a spinal cord injury, the upper extremities play a more important role, not only in propelling the wheelchair, but also in performing other activities such as transfers. This requires more frequent use and more loads on these extremities.

Previous studies showed high prevalence of upper limb pain on the shoulder joint, as well as the wrist and elbow joints [3,4,5,6,7]. Nevertheless, the etiology of the pain is not clear yet. It may be due to repetitive movements performed or a more frequent use of the upper limb to perform the activities already mentioned: wheelchair propulsion and transfers. In fact, the long-term use of the wheelchair is a biomechanical challenge due to the fact that the upper limb is not optimized to support repetitive movements contrary to what the lower extremity does during the gait. In [8], the impact of the type of propulsion technique on the development of shoulder injuries is further studied, encouraging to identify properties of the technique that reduce biomechanical loads on the shoulder joint. In [9], skills to improve the propulsion techniques are shown after a randomized test with 106 veterans which identified the improvements obtained after a specific wheelchair propulsion training program. Moreover, the Clinical Practice Guideline for Preservation of Upper Limb Function following spinal cord injury describes the different factors that have an impact on developing shoulder injuries [10]. These factors combine aspects such as propulsion techniques, assistance during transfers and wheelchair type and configuration [11].

The impact of wheelchair type and configuration has been studied from different points of views: weight, configuration or typical usage [12,13,14,15,16,17,18]. The objective, in all cases, is to unveil the relationship between these factors and the risk of lesions on the patients. These studies focus on physiological outcomes such as cardio-respiratory measures (systolic and diastolic blood pressure), oxygen and carbon dioxide in pulmonary ventilation. There are studies which show a difference in tetraplegic patients when a lightweight or ultralight wheelchairs are used [12,18]. Unfortunately, they are mainly based on physiological data. The main conclusion of these studies is that customization of the wheelchair may diminish the risk of further problems such as respiratory complications or shoulder injuries in patients with spinal cord injury [19,20]. Amongst the main aspects that may have an impact during the wheelchair propulsion are: the size of the seat, the shape and angle of the seat, the feet support, the height of the back, the shape and angle of the back, the arm supports, the size of wheels, the angle of the wheels and of course, the structure and material of the wheelchair. Several studies focus on the ergonomics and position in different wheelchairs [20,21]. However, few studies specifically address the kinematic and kinetic affects of wheelchair configuration and the biomechanics of the upper limb joints. Ref. [13] showed no difference on the kinematics of the upper limb when the weight of the wheelchair is changed, maintaining the configuration. A kinetic study was deemed as neccesary to better understand the impact of wheelchair weight on the upper limb joints. Basically, the weight of the wheelchair seems to have more relevance when the lesion is higher (i.e., more impact on tetraplegic users than on paraplegics). This fact does not only impact on the joint loads of the upper limb, but also on the speed and distance. Moreover, it influences physiological aspects such as heart rate coming from the effort made. Nevertheless, there is a gap in combining kinetic and kinematic analysis and therefore, a lack of evidence of the impact of wheelchair used on the upper limb joint biomechanics.

Gait analysis is well established in clinical practice through a great variety of clinical applications integrated in healthcare centers and hospitals to track the state of different illneses [22,23]. For the upper limb, there is not a comprehensive and user-friendly application which could support the motion analysis of the upper limb by clinicians in order to prescribe wheelchairs through personalization. Therefore, this paper focuses on developing a new systematic procedure and methology to support the analysis of the impact of the wheelchair configuration providing a very comprehensive study on this field. Moreover, it compares kinematic and kinetic variables of the upper limb joints when propelling distinct wheelchairs with different characteristics.

This methodology is used to analyze the differences of two groups of patients, tetraplegics and paraplegics. The structure of the paper is as follows. The metholody created is presented in Section 2. Section 3 describes the results of the applying this methodolody in an experimental test. In Section 4 an analysis of the results obtained is presented, and the paper is concluded in Section 5.

## 2. Materials and Methods

### 2.1. Methodology

**Figure 1:**
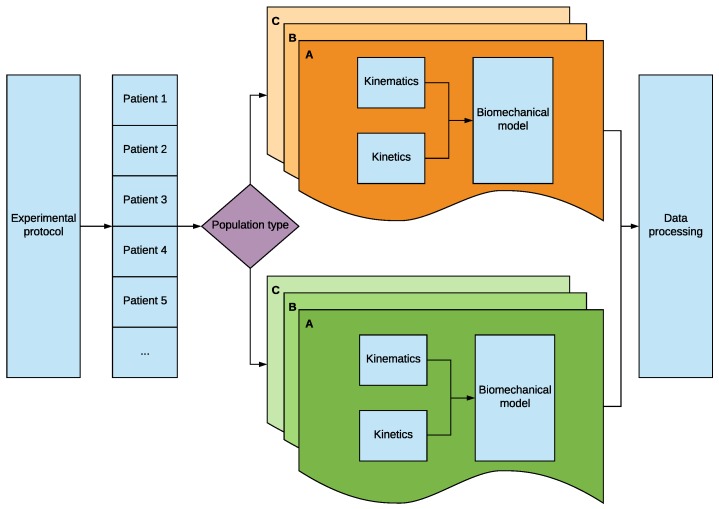
Diagram of the methodology developed which allows to study different patient populations using several wheelchairs.

A methodology which combines kinetic and kinematic data during wheelchair propulsion has been created including a tailored-made biomechanical model for the upper limb. It includes all the steps required by a clinician to accomplish a propulsion study in a fast and efficient manner. Moreover, it has been implemented in an automated application (https://github.com/Robolabo/UpperLimbMotion). The process developed in this paper is shown in Figure 1. This process is a practical application of the designed methodology which allows the study of the upper-limb behavior of different populations (e.g., *X*,*Y*) when using different wheelchairs. In Figure 1, different wheelchairs are represented by letters (e.g., A, B, C). For example, population *X* (orange) is the paraplegics and population *Y* (green) the tetraplegics; whereas letters A, B and C refer to different wheelchair models used in a concrete experimental protocol. Before starting the study, the definition of an experimental protocol must be set. Later on, the population to perform such protocol is selected, informed and classified. Afterwards, the populations to compare must be classified, in order to gather data separately. The test performed must be repeated with as many wheelchairs as required by the experimental protocol. The order in which the test must be performed will depend on the aim of the study; nevertheless, this methodology allows to perform ordered and randomized tests. Furthermore, a clinical study in form of a randomized controlled trial may be set in which patients are allocated in groups and compression forces at the shoulder and wrist can be set as main variables. Finally, all the variables must be analyzed in an automated process which computes and shows all the relevant data. In this paper, the study of the upper-limb of two populations: paraplegics and tetraplegics is carried out when using two different wheelchairs. In what follows, the different stages of the procedure are described.

### 2.2. Experimental Protocol

The study took place at the Biomechanics and Assistive Technology Unit of the National Hospital for Paraplegics in Toledo (Spain). The sessions lasted 30 min each in which the participants propelled a wheelchair at a speed of 0.833 m/s (3 km/h) on a level treadmill. Each session began with a three minutes warm-up exercise followed by a one minute break. No previous training was done with any of the two wheelchairs used. Then, the participant propelled one wheelchair using a semicircular propulsion pattern for three minutes, had a one minute break and repeated this exercise three times. This first exercise lasted 15 min and it was repeated with two wheelchairs in the same manner, completing the 30 min session. The wheelchairs used were: wheelchair A, the Action 3 model from Invacare [24] and wheelchair B, the Ventus model from Ottobock [25]. The main characteristics of each of the wheelchairs are shown in Table 1.

For safety purposes, the participants were monitored during the exercise by measuring the oxygen saturation and the heart rate. Due to the nature of being a submaximal exercise, there was no need to set a specific HR threshold. Nevertheless, the clinical staff of the Hospital suggested from previous experimentations to stop in the case that 130 bpm were achieved.

### 2.3. Participants

Ten people with spinal cord injury from the Hospital participated in this study with mean age of 41.1 ± 13.2 years, mean weight of 72.1 ± 5.4 kg and mean height of 1.76 ± 0.08 m. The characteristics of the wheelchairs that the participants own are shown in Table 2. This shows that there are no significant differences between the wheelchairs used in the experimental protocol and the ones the participants own, except for patient P06. Therefore, it was considered that a training with the wheelchairs would not have in impact on the forces obtained. Consequently, it was decided not to perform a training session with the two models used for the experimental protocol. All participants provided a signed informed consent after the test was explained and any concerns were discussed before the sessions started. The study was approved by the Local Ethics Committee of the Hospital (application reference number 27092012-74). Criteria for inclusion were: people above 18 years old, with a lesion over four months that propelled a manual wheelchair. Exclusion criteria were: surgical procedures on the upper-limb, current pain or any medical situation against moderate exercise. Two groups shown in Table 3 were classified based on the level of injury: one group had five participants with thoracic lesions, tetraplegics, and another one of five patients with cervical lesions, paraplegics. To classify the sensor and motor information, the American Spinal Injury Association (A.S.I.A.) scale is used [26].

### 2.4. Kinematics

The kinematic data collection was performed using the Kinescan system provided by the Instituto de Biomecánica de Valencia (IBV) [27]. This system allows the analysis of motion in real time and it has the following components: four pulnix digital cameras which recorded the tests at a 50 Hz frequency, infrared optics integrated into the cameras, infrared technology torches (also integrated along with the cameras), a set of reflective markers and a license to use the Kinescan software. The digital cameras allow to record at a maximum frequency of 100 Hz. Because of computational limitations in the set-up, a frequency of 50 Hz was selected. The Kinescan software allows to set the parameters to capture, the frequency, variables to compute, images in real time and visualization of the results in 3D. The data obtained from the 3D motion captured by the kinescan system is included as input into the inverse dynamic model [28].

### 2.5. Kinetics

The kinetic information was captured using two SmartWheels, one in each side [29]. These wheels have strain gauges and encoders to capture the forces on the hand-rim during propulsion. A synchronization from the Kinescan software to trigger the start of both kinetic and kinematic data is set. Smartwheels recoded data at 240 Hz. This data was filtered by using a fourth order Butterworth low-pass filter with a cutoff frequency of 20 Hz and a zero phase lag as already described in [28]. The moment in which the patient holds and releases the push-rim of the wheelchair is identified when the moment at the push-rim is higher or lower than 1 Nm respectively [6,30].

### 2.6. Biomechanical Model

**Figure 2:**
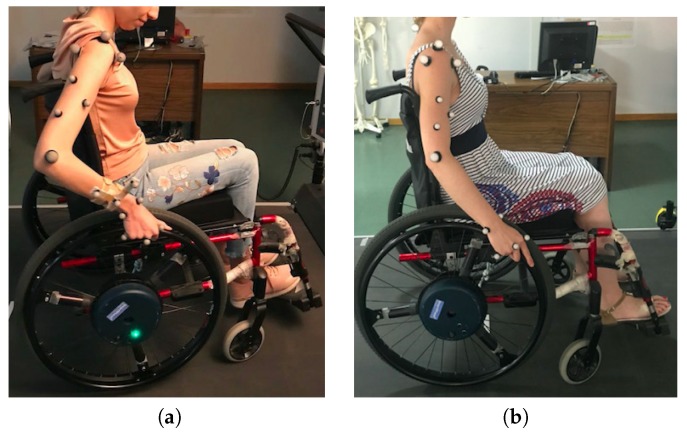
(**a**) Initial and (**b**) final configuration of markers. (**a**) Three reference markers were included in the forearm to act as reference for the markers of the hand and the elbow as any marker needs a reference system with three markers; (**b**) The forearm reference markers were removed by referencing the markers of the elbow to the reference markers of the arm and the ones of the hand to the reference system of the hand itself.

An inverse dynamic model was defined to calculate the positions, forces and moments that each of the joints of the arm are supporting [28]. By following the recommendations of the International Society of Biomechanics (ISB) [31], the local reference systems for all the joints of the upper limb were set. The model is previously described in [28]. However, the number of reflective markers were reduced from the original model: from 18 to 15 markers. This reduction has been done by referencing the markers of the hand to the hand reference system and the markers of the elbow to the three reference markers of the arm. Therefore, the reference markers of the forearm were removed, as shown in Figure 2. The rest of the markers were located at the bone protrusions.

This biomechanical model relies on anthropometric data of the patients and body segments are considered as rigid bodies with revolution geometries [28]. The segments of the arm defined are shown in Table 4.

The shoulder is considered one of the most complex joints of the human body. There are five different joints within the shoulder: glenohumeral, sternocalvicular, acromioclavicular, coracoclavicular, and the scapulothoracic joint. The glenohumeral joint is the major one, being the union between the head of the humerus and the scapula conforming a ball and socket joint. It is the joint that provides more movements in the body as it allows flexion, extension, hyperextension, abduction, adduction, horizontal abduction and adduction, and medial and lateral rotation of the humerus [32]. Taking into account the movements preformed by the shoulder when propelling a wheelchair, the glenohumeral joint was the one modeled. The elbow is a simpler joint but it also has its complexity as it connects three different joints which are the humeroulnar, humeroradial and proximal radioulnar joints [32]. Finally, the wrist includes mainly the radiocarpal and the intercarpal joints which consist of a number of small and individual joints. All these connections make the wrist to be a complex joint. Most of the wrist motion happens in the radiocarpal joint, in which the radius connects with the bones and the one in which the model focuses [33]. Therefore, the model takes into account this musculoskeletal system by defining the three different segments shown in Table 4, trunk, arm and hand.

### 2.7. Data Processing

**Figure 3:**
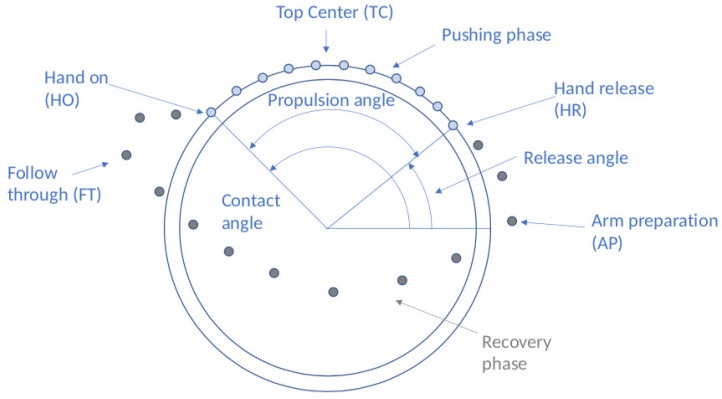
Propulsion cycle with main contact points and angles. Points represent a discretized position of the hand on the different phases. Pushing phase on the ring in blue and recovery phase in the free space in grey.

From the recorded data, the most stable and representative minute, without accelerating and decelerating components, was extracted. The extraction of this minute is done following the same procedure among all participants and wheelchair types. From this minute, five consecutive cycles were selected and normalized. Figure 3 shows a single cycle. The process of selecting the most representative minute is done manually after the video recording of the movement and before digitizing the videos. Therefore, the values obtained for that minute are the ones included in the data processing software. The normalization of the five consecutive cycles is done automatically by the software developed. Furthermore, the automated application has been developed in which the data processing software is embedded and allows to perform a biomechanical study in a fast and efficient manner.

**Figure 4:**
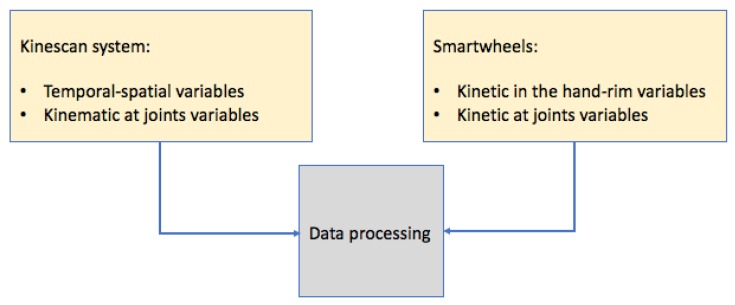
Data gathering.

The application was developed to compute all the variables shown in Table 5. These variables provide a comprehensive approach combining most of all the important variables during wheelchair propulsion. Other variables such as the energy expenditure, cadence, rate of rise and the kinemtaics during the kinematics during the recovery phase at all joints could be included. The kinescan system and the smartwheels provide the data that is processed in a customized developed software in MATLAB, as shown in Figure 4.

The temporal spatial variables provide information related to the propulsion style. The kinetic variables in the hand-rim provide information about forces and moments that are produced in the hand-rim. It includes the effective force which provides a rate between the tangential and the total force exerted during propulsion. Moreover, the positions of all the joints are obtained. These variables are calculated using inverse kinematics and the Euler angles. For all these variables (temporal–spatial, kinematics and kinetics), the maximum and minimum values during the propulsion cycle are obtained, the range of motion (ROM) and the value in the following important moments of the propulsion: when the hand contacts the hand-rim (HO: hand on), at the top position of the hand-rim (TC: top center), when the hand losses contact with the hand-rim (HR: hand release), at the moment in which the hand has reached the most anterior point within the propulsion cycle (FT: follow through) and when the hand is at the most posterior point (AR: arm preparation). Finally the kinetic variables at the joints are obtained. Furthermore, the maximum and minimum values are obtained as well as the forces and moments in the important instants of the propulsion already explained.

The laboratory reference system is set-up in such a way that the *x*-axis corresponds to the movement direction, the *y*-axis corresponds to the lateral movement (positive to the left, negative to the right) and the *z*-axis corresponds to the vertical movement. Therefore, the forces and moments with positive and negative values have the following meaning:

- $F_{x}$: + anterior, − posterior
- $F_{y}$: + lateral, − medial
- $F_{z}$: + superior, − inferior
- $M_{x}$: + adduction, − abduction (+ cubital, − radial for the wrist)
- $M_{y}$: + flexion, − extension
- $M_{z}$: + pronation, − supination (+ internal rotation, − external rotation for shoulder and wrist)

### 2.8. Statistical Analysis

As the sample is reduced (ten participants in total), a non parametric test was chosen to perform the statistical analysis as data normality could not be ensured. The Wilcoxon Rank-Sum test [34] has been used to compare two samples: one sample with all the participants propelling wheelchair A and another one propelling the wheelchair B. This test examines whether the populations differ in median being the null hypothesis that both samples are identical with a significance level of p<0.05.

## 3. Results

The whole methodology developed was tested with the experimental protocol explained. Except for one patient which test with wheelchair A could not be completed, the rest of the participants successfully completed the test. By using the two wheelchairs presented, a statistical analysis of differences between paraplegics and tetraplegics is performed. Differences exist between the two groups coming from the different type of lesions, but these differences are affected also by the type of wheelchair used [35,36].

### 3.1. Impact of the Wheelchair on Temporal-Spatial and Kinetic Variables in the Hand-Rim

With respect to the temporal–spatial variables shown in Table 6, no differences that show significance between paraplegic and tetraplegic groups are found; therefore, there is no impact coming from the wheelchair used.

With respect to the kinetic variables in the hand-rim shown in Table 7, there is a difference between paraplegics and tetraplegics in the maximum total force applied and this difference appears in the same manner with both wheelchairs. In most of the cases, the value of the force and moment applied is always higher in patients with tetraplegia.

### 3.2. Impact of the Wheelchair on the Shoulder

**Figure 5:**
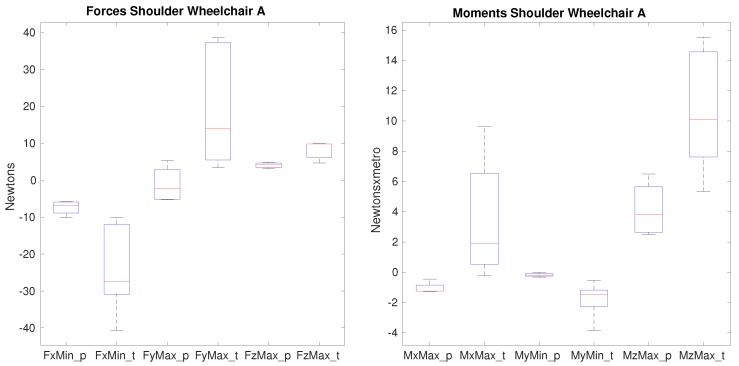
Forces and moments that show significance on the shoulder between paraplegics and tetraplegics when propelling wheelchair A. FxMin represents the minimum value of posterior force on the shoulder, FyMax shows the maximum lateral force on the shoulder, FzMax represents the maximum superior force. MxMax shows the maximum value of the adduction moment, MyMin the minimum value of the extension moment and MzMax the maximum value of the internal rotation moment on the shoulder. In all cases, the values for paraplegics (_p) and tetraplegics (_t) are shown.

Differences between paraplegic and tetraplegic patients are impacted by the wheelchair used (see Table 8). When propelling wheelchair A, there are important differences with significance (p<0.05) in the forces in all the axes: *x*, *y* and *z*, as shown in Figure 5.

**Figure 6:**
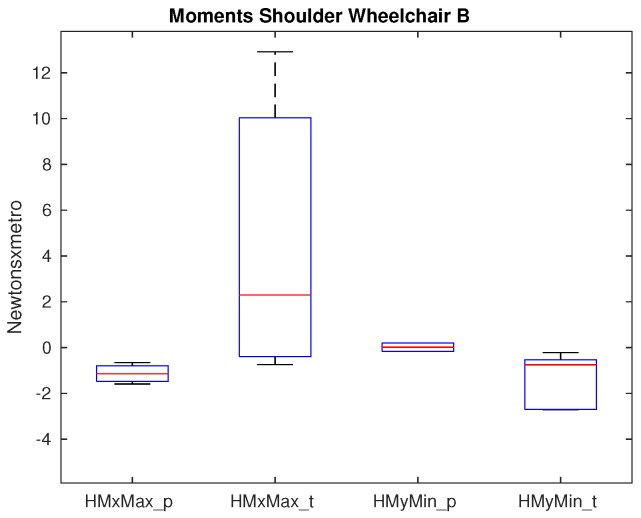
Moments that show significance on the shoulder between paraplegics and tetraplegics when propelling wheelchair B. HMxMax shows the maximum adduction moment on the shoulder and HMyMin shows the minimum extension moment on the shoulder. In all cases, the values for paraplegics (_p) and tetraplegics (_t) are shown.

The minimum posterior force, the maximum lateral force and also the maximum superior force are significantly different when wheelchair A is used. Not only the medians are different but also the range of values is much wider in the case of tetraplegic patients representing a high variability. The median of the minimum posterior force increases from −6.8 N when paraplegics propel the wheelchair A to approximately −28 N in case of tetraplegics. The median of the maximum lateral force differs from −2.2 N when paraplegics propel the wheelchair to 13.9 N when tetraplegics do. The median of the maximum superior force between paraplegics and tetraplegics is lower, 4.2 N when paraplegics propel and 9.8 N when tetraplegics do, but still significance is observed. With respect to the moments, there is also important significance at this joint in all axes: the maximum adduction moment in which the median value is −1.25 Nm when paraplegics propel the wheelchair and 1.9 Nm when tetraplegics do. The median minimum extension moment varies from −0.18 Nm for paraplegics to −1.5 Nm for tetraplegics. The median maximum internal rotation moment is 3.8 Nm when paraplegics propel and 10 Nm when tetraplegics do. Differences are not as high as the ones obtained for the forces but still significance is obtained. When using wheelchair B, there is no significance on the force that the shoulder is supporting, only the maximum lateral force nearly shows significance (p=0.064), being the only variable of the shoulder that is close to the established significance level. With respect to the moments, the significance of the internal rotation moment disappears, as shown in Figure 6.

Nevertheless, with respect to the kinematic variables, there are no differences on the shoulder impacted by the use of two different wheelchairs. In Table 9, only the results of the maximum and minimum values as well as the rage of motion (ROM) are shown, as a representative sample on how the shoulder behaved during this test. In general, the values are higher for tetraplegics and the ROM is usually higher in tetraplegics as well. However, this difference does not show a statistical difference when different wheelchairs are used.

### 3.3. Impact of the Wheelchair on the Elbow

**Figure 7:**
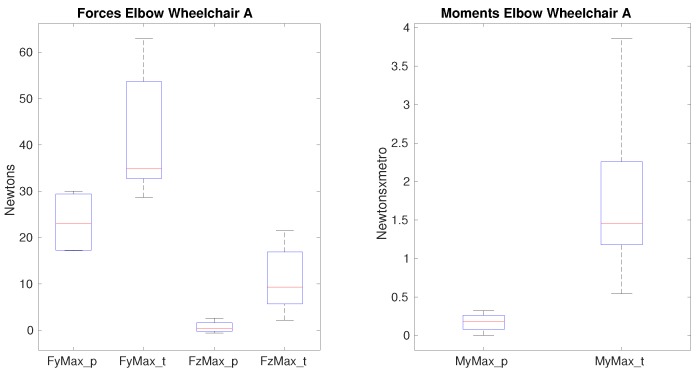
Forces and moments that show significance on the elbow between paraplegics and tetraplegics when propelling wheelchair A. FyMax represents the maximum value of lateral force on the elbow, FzMax shows the maximum superior force on the elbow. MyMax shows the maximum value of the flexion moment. In all cases, the values for paraplegics (_p) and tetraplegics (_t) are shown.

Differences between paraplegic and tetraplegic patients on the maximum lateral force, on the maximum vertical force and the maximum flexion moment when propelling the wheelchair A (see Table 10) were found. As shown in Figure 7, the median value of the maximum lateral force varies from 23 N for paraplegics to almost 35 N for tetraplegics; the median of the maximum vertical force shows a higher difference as the value for paraplegics is 0.37 N whereas for tetraplegics this value is 9.3 N. With respect to the median of he maximum flexion moment, this value is almost 10 times bigger for tetraplegics which value is 1.4 Nm for paraplegics and 0.18 Nm for tetraplegics.

**Figure 8:**
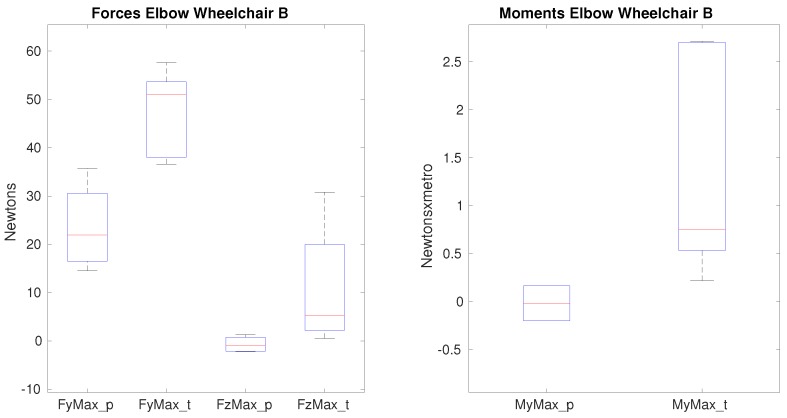
Forces and moments that show significance on the elbow between paraplegics and tetraplegics when propelling wheelchair B. FyMax represents the maximum value of lateral force on the elbow, FzMax shows the maximum superior force on the elbow. MyMax shows the maximum value of the flexion moment. In all cases, the values for paraplegics (_p) and tetraplegics (_t) are shown.

Nevertheless, on this joint there is no difference impacted by the use of a different wheelchair. In Figure 8, the same forces and moments present significance. In both scenarios the forces and moments present significance between the two groups of patients but such differences are similar when a different wheelchair is used. However, the range of values obtained when propelling wheelchair B presents less variability and therefore they are more stable.

With respect to the kinematic variables, there are no differences in shoulder impact by the use of two different wheelchairs. In Table 11, only the results of the maximum and minimum values as well as the rage of motion (ROM) are shown, as a representative sample on how the shoulder behaved during this test. In general, the values are higher for tetraplegics and the ROM is usually higher in tetraplegics as well. However, this difference is not significant when different wheelchairs are used.

### 3.4. Impact of the Wheelchair on the Wrist

**Figure 9:**
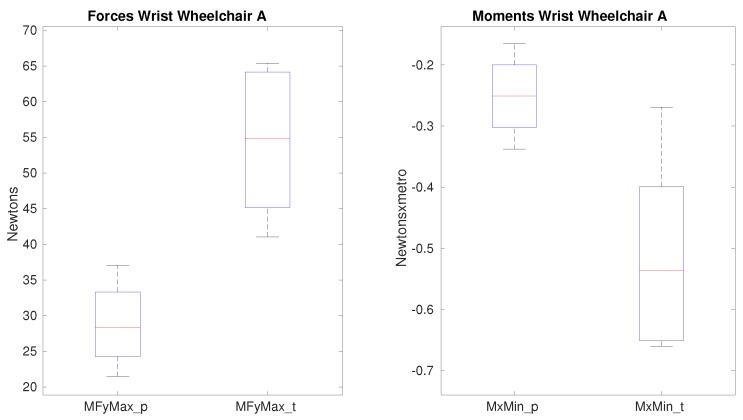
Forces and moments that show significance on the wrist between paraplegics and tetraplegics when propelling wheelchair A. MFyMax shows the maximum lateral force and MxMin shows the minimum radial moment on the wrist. In all cases, the values for paraplegics (_p) and tetraplegics (_t) are shown.

Between paraplegic and tetraplegic patients (see Table 12) on the lateral force and the radial moment as shown in Figure 9 when wheelchair A is used. In the case of the force, the medians are completely different from approximately 30 N when the paraplegics propel the wheelchair to almost 55 N in case of the tetraplegics. For the radial moment the difference is not so high, −0.25 Nm for paraplegics and −0.54 Nm for tetraplegics but a significance of p=0.0317 is obtained.

**Figure 10:**
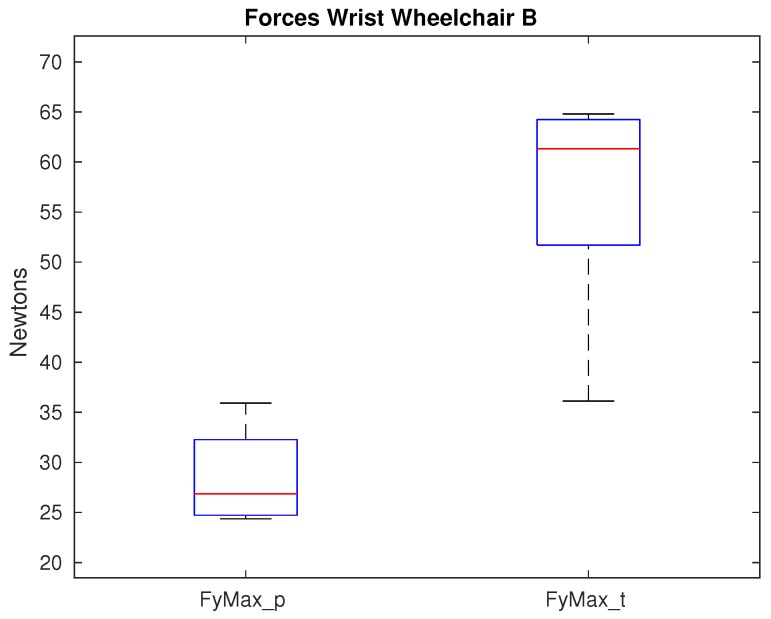
Maximum lateral force that shows significance on the wrist between paraplegics and tetraplegics when propelling wheelchair B. Values for paraplegics (_p) and tetraplegics (_t) are shown.

Nevertheless, when wheelchair B is used, only the lateral force shows significance as observed in Figure 10. For the rest of the kinetic variables, the range of the median values is similar to the ones obtained with wheelchair A, but the quartile values are more reduced when the model B is used. Therefore, more stability is provided on this joint when the model B is used.

With respect to the kinematic variables, even though there are differences as shown in Table 13, no statistical difference has been observed from the use of the two wheelchair models. Only the ulnar-radial and flexo-extension movements are shown as they are the most representatives on the wrist.

## 4. Discussion

The goal of this study was to present and use a new developed methodology to show differences in all relevant propulsion variables when different wheelchairs are used. The main contribution of this study is that most of all representative variables are included: temporal–spatial, kinetics in the hand-rim and kinematics and kinetics of the joints of the upper limb. Moreover, this whole analysis is performed under a new methodology approach which provides automation and effectiveness to this study. In addition, it could be extrapolated to other propulsion studies. For example, studying the shoulder consequences while propelling the same wheelchair during long time periods, analyzing the differences in the upper-limb when propelling the same wheelchair model in different inclinations, or studying the impact of adding weight to a wheelchair. This is possible because the methodology to apply is the same, only the conditions to test are different. The main advantage of this new methodology is that a clear protocol and procedure to follow are set. Moreover, this analysis is conducted under a new approach which provides automation and effectiveness to this kind of studies. Our process could be extrapolated to other propulsion-related studies (i.e., investigating the shoulder alterations while propelling the same wheelchair during long time periods, the upper-limb alterations when propelling in different inclinations or studying the impact of adding weight to a wheelchair). It is obvious that the methodology to apply is the same, only the testing conditions differ. The main advantage of our approach is that both a clear protocol and a procedure to follow are set which allows obtaining biomechanical data in a fully automated manner. Along with the software, a comprehensive documentation is also provided, explaining in detail all the relevant process underlying our methodological approach: marker placement in the arm of the patient, movement capture with the Kinescan system, synchronization of the SmartWheels with Kinescan, marker analysis, kinematic and kinetic model equations, and data averaging and analysis. Furthermore, in this process all the kinematic and kinetic variables are embedded providing the option to make comprehensive results available that could support different wheelchair propulsion studies. This detailed methodology allows the Hospital to follow the very same protocol for all patients, keeping consistency between different patients and trials.

### 4.1. Impact of the Wheelchair on Temporal–Spatial and Kinetic Variables in the Hand-Rim

It is expected that differences between paraplegic and tetraplegic patients exist when propelling a manual wheelchair coming from the lesion. Actually, even though there are no differences which show significance, there are some studies in the literature that state that an increase of contact angle and a decrease in push frequency are two important variables that have an impact on pain on the upper-limb [8]. From our study, when paraplegics propel both wheelchairs, the push frequency is lower when a lighter wheelchair is used (Table 6); nevertheless, for tetraplegics, three out of the five patients increased the push frequency when a lighter wheelchair is used. For the contact angle, the value is higher for paraplegics and the impact of the different wheelchairs is almost not perceived. Therefore, there is no impact on the temporal–spatial and kinetic variables in the hand-rim derived from the use of the two different wheelchairs.

### 4.2. Impact of the Wheelchair on the Shoulder

The differences that appear between paraplegic and tetraplegic patients on the shoulder show that higher force values are obtained for tetraplegics when using the wheelchair A. This coincides with previous studies [37,38]. In case of anterior forces, tetraplegics show higher values (21% in average) but it is in the posterior forces in which this difference increases: in average, the value is −7 N for paraplegics and −23 N for tetraplegics. When the lateral force is analyzed, paraplegics show a reduced medial force and tetraplegics a high lateral one. It shows that the force on this joint goes into a completely different direction. Finally, when the superior and inferior force is analyzed, the superior force is double for tetraplegics (4 N and 8 N respectively). The inferior force is quite similar. For the moments, on the adduction-abduction moment, the direction is different being abduction for paraplegics and adduction for tetraplegics, showing significance with an increase of 68% of the value. The extension moment in average is 10 times higher for tetraplegics (−1.8 Nm compared with −0.17 Nm obtained for paraplegics) and the internal rotation in average is 2.5 times higher (10.7 Nm compared with the 4.2 obtained for paraplegics). This analysis reinforces the fact that tetraplegic patients may report higher shoulder pain due to higher shoulder forces and moments developed during propulsion [39]. Besides, the tetraplegic group had more individuals with complete injuries than in the paraplegic one, which may also bias the correlation between tetraplegia and shoulder pain. Future studies should consider the possible relationships underlying type of injury –mainly motor-complete versus incomplete- with regards to shoulder kinetics and reported pain [40,41]. The fact of using a different wheelchair which has a different configuration and weight has an impact as stated in the Clinical Practice Guidelines for preservation of upper limb function following spinal cord injury [10]. In our study, this impact is clearly shown, as no difference appears between paraplegic and tetraplegic patients when using wheelchair B. This is a huge impact taking into account the important differences observed when wheelchair A is used. For the moments, there are also differences that show significance even though the values are in a similar range to the ones obtained with wheelchair A.

### 4.3. Impact of the Wheelchair on the Elbow

In general, most of the forces that the elbow is supporting are higher for tetraplegic patients; in average the increase is around 3%. The vertical force is the one which shows the biggest difference being 6.8 N for paraplegics and 11 N for tetraplegics obtaining significance. The impact of using a different wheelchair is not shown for the forces nor for the moments, which values are quite similar as shown in Figure 7 and Figure 8. Actually, the differences were spare and small, so body weight and the other factors –mainly slight geometric configurations- does not make such difference in the biomechanical variables. These results are consistent with different studies which show that the prevalence of the upper-limb pain exists, mainly in the shoulder and the wrist with a more reduced impact on the elbow [3,4,5].

### 4.4. Impact of the Wheelchair on the Wrist

The values obtained for the forces on the wrist are always higher for tetraplegics showing statistical significance on the lateral force. When comparing the maximum force values using both wheelchairs, the difference between paraplegics and tetraplegics of the maximum forces in average are higher when using a lighter wheelchair. Specially this difference is observed in the superior force, in which the average difference in the superior force is 5.6 N between tetraplegics and paraplegics and 12.4 N when using a lighter wheelchair. Therefore, a lighter wheelchair does not entail a positive impact on tetraplegics on the wrist. Nevertheless, on the difference between the minimum forces, a lighter wheelchair impacts reducing it and therefore, impacting positively on the tetraplegics patients having, in average, lower minimum force values than compared to the paraplegic patients. The fact that wheelchair B has a positive impact on minimum force values but negative on maximum force ones, makes difficult to analyze the real impact on the wrist based on the two wheelchairs. Previous investigations [42,43] show a direct relationship between the push-rim force and the wheelchair configuration with the carpal tunnel syndrome. In our study, no significance was found on the push-rim force (Table 7) coming from the use of different wheelchair, which explains the result obtained and supports the fact that this study is not totally conclusive on the wrist. Therefore, further analysis should be done.

### 4.5. Future Directions

The impact of the wheelchair configuration should be tested in an extensive way to raise conclusions. Future studies should include more patients for both groups: paraplegics and tetraplegics with different levels of lesion in order to understand the impact of wheelchair type upon lesion characteristics. Moreover, more advanced and comprehensive biomechanical model of the shoulder is desirable. Furthermore, the natural propulsion patterns of the participants should be taken into consideration in future works as it may impact on the results.

## 5. Conclusions

This study proved that the methodology proposed is appropriate to accomplish kinematic and kinetic studies on different populations of spinal cord injury patients. The experimental protocol was easy to follow, and the application developed was easy to use by clinicians. This methodology supported experimentation and automated data collection and analysis. The experimental protocol conducted allowed us to confirm that the methodology proposed fulfills the requirements and can be used within a clinical setting without specialized technical support. Besides, the results obtained from the experiments confirm that upper-limb biomechanical load is related with lesion level and type/characteristics of the wheelchair. It has been observed that the shoulder of tetraplegic patients behaves similarly to the paraplegic patients when using a lighter wheelchair, which shows a lower tendency to develop upper-limb lesions. This observation aligns with previous reported work, such as [12,18]. We did not find significant differences on the remaining joints analyzed with respect to the wheelchair used. Our results are therefore aligned with preliminary findings on the relationship between wheelchair type and upper-limb depending on the lesion characteristic. Nonetheless, further investigations need to be done to fully automate the process, including automated group-averaging and statistical analysis, in order to fully support this kind of experimental studies. Also, making the study with participants with the same level of injury will provide more robust results. Therefore, the proposed methodology could be used to accomplish these investigations.

## Figures and Tables

**Table 1:** Wheelchairs (WCh) used for the experimental protocol.

Characteristics	WCh A	WCh B
Total height (cm)	97.0	92.0
Total width (cm)	67.0	60.5
Total depth (cm)	95.0	88.0
Seat-floor height (cm)	48.0	44.0
Seat-footrest height (cm)	48.0	40.0
Seat height (cm)	43.0	38.0
Seat width (cm)	42.0	40.0
Seat depth (cm)	40.0	38.0
Seat inclination (${}^{\circ }$)	4.1	7.5
Weight (kg)	13.0	11.0
Camber (${}^{\circ }$)	0	0
Wheel diameters (mm)	600	600

**Table 2:** Characteristics of the wheelchairs (WCh) of the participants.

Participants	WCh Model	WCh Weight (kg)	WCh Seat Width (cm)	WCh Seat Depth (cm)
P01	TiLite ZRA	9	36	38
P02	Oracing	9	41	43
P03	Kuschall K-4	9	40	41
P04	Action 3	13	40	41
P05	Action 3	13	40	41
P06	Panthera	5.5	41	35
P07	Kuschall Champion	10	38	38
P08	RGK Tiga	10	37	37
P09	Action 3	13	42	42
P10	Action 3	13	40	40

**Table 3:** Participants lesion characteristics.

Participants	Gender	Lesion Level	Time Since Injury (Months)	A.S.I.A.	Type of Lesion	Group	
P01	F	T6	28	A	Complete	Paraplegic	(P)
P02	M	T11	5	C	Incomplete	Paraplegic	(P)
P03	M	T1	555	A	Complete	Paraplegic	(P)
P04	F	T7	7	B	Incomplete	Paraplegic	(P)
P05	M	T5	5	A	Complete	Paraplegic	(P)
P06	M	C6-7	285	A	Complete	Tetraplegic	(T)
P07	M	C8	59	A	Complete	Tetraplegic	(T)
P08	M	C4	15	D	Incomplete	Tetraplegic	(T)
P09	M	C6-7	349	A	Complete	Tetraplegic	(T)
P10	M	C8	472	A	Complete	Tetraplegic	(T)

**Table 4:** Biomechanical model.

Acronym	Description	Segment
c7	Seventh cervical vertebra	Trunk
acrr	Right acromion-clavicular bone protrusion	Trunk
acrl	Left acromion-clavicular bone protrusion	Trunk
hha	Anterior point of humeral head	Trunk
hhp	Posterior point of humeral head	Trunk
rm1	Reference marker on the arm number 1	Arm
rm2	Reference marker on the arm number 2	Arm
rm3	Reference marker on the arm number 3	Arm
epc	External epicondyle	Arm
ipc	Internal epicondyle	Arm
ulr	Ulnar styloid	Hand
rdl	Radial styloid	Hand
2m	Second metacarpus	Hand
3m	Third metacarpus	Hand
5m	Fifth metacarpus	Hand

**Table 5:** All variables analyzed in the procedure proposed divided in groups.

Type of Variable	Description	Equation/Specifications
Temporal–spatial	Cadence: number of strokes per second	PF (st/s)
	Pushing phase: time elapsed since the hand-rim is held until it is released	$P_{phase}$ (s)
	Recovery phase: times elapsed since the hand-rim is released until it is held again	$R_{phase}$ (s)
	Quotient between the pushing and recovery phase	$P_{phase}/R_{phase}$
	Distance covered in a propulsion cycle	Dist (m)
	Contact angle: angle at which the hand-rim is held	CA (${}^{\circ }$)
	Release angle: angle at which the hand-rim is released	RA (${}^{\circ }$)
Kinetic in the hand-rim	Force in the reference system of the hand-rim	$F_{SW,x},F_{SW,y},F_{SW,z}$ (N)
	Maximum total force in the hand-rim	$F_{tot}=\sqrt{F_{SW,x}^{2}+F_{SW,y}^{2}+F_{SW,z}^{2}}$ (N)
	Maximum tangential force in the hand-rim	$F_{tang}=-F_{SW,x}sin\theta +F_{SW,y}cos\theta$ (N)
	Effective force	$F_{eff}=\frac{F_{tang}}{\sqrt{F_{SW,x}^{2}+F_{SW,y}^{2}+F_{SW,z}^{2}}}$
	Elevation rate of the total force	$ERF=\frac{dF_{tot}}{dt}$ (N/s)
	Moments in the reference system of the hand-rim	$M_{SW,x},M_{SW,y},M_{SW,z}$ (Nm)
	Maximum total moment in the hand-rim	$M_{tot}=\sqrt{M_{SW,x}^{2}+M_{SW,y}^{2}+M_{SW,z}^{2}}$ (Nm)
	Elevation rate of the total moment	$ERM=\frac{dM_{tot}}{dt}$ (Nm/s)
Kinematic at joints (Value of joint’s position along the whole propulsion cycle: pushing and recovery phases)	Shoulder elevation: adduction-abduction	$\alpha_{sh}$(${}^{\circ }$)
	Shoulder elevation plane: flexion-extension	$\beta_{sh}$ (${}^{\circ }$)
	Shoulder internal rotation	$\gamma_{sh}$ (${}^{\circ }$)
	Elbow internal rotation	$\beta_{e}$ (${}^{\circ }$)
	Elbow flexion-extension	$\gamma_{e}$ (${}^{\circ }$)
	Wrist ulnar-radial deviation	$\alpha_{wr}$ (${}^{\circ }$)
	Wrist internal rotation	$\beta_{wr}$ (${}^{\circ }$)
	Wrist flexion-extension	$\gamma_{wr}$ (${}^{\circ }$)
Kinetic at joints (Value of joint’s forces and moments along the whole propulsion cycle: pushing and recovery phases)	Maximum and minimum forces	$F_{x},F_{y},F_{z}$ (N)
	Forces in HC, TC, HO, FT and AR	$F_{x},F_{y},F_{z}$ (N)
	Maximum and minimum moments	$M_{x},M_{y},M_{z}$ (Nm)
	Forces in HC, TC, HO, FT and AR	$M_{x},M_{y},M_{z}$ (Nm)

**Table 6:** Temporal–spatial variables (mean ±SD).

	Paraplegic Group		Tetraplegic Group	
	WCh A	WCh B	WCh A	WCh B
PF (st/s)	1.25 ±0.43	1.04 ±0.20	1.05 ±0.28	1.14 ±0.30
Dist (m)	0.72 ±0.26	0.82 ±0.17	0.83 ±0.26	0.77 ±0.24
$P_{phase}$ (s)	0.34 ±0.11	0.41 ±0.07	0.44 ±0.13	0.43 ±0.14
$R_{phase}$ (s)	0.55 ±0.23	0.59 ±0.17	0.57 ±0.18	0.50 ±0.15
$P_{phase}/R_{phase}$	0.65 ±0.09	0.71 ±0.09	0.80 ±0.11	0.86 ±0.07
CA (${}^{\circ }$)	113.86 ±2.40	111.27 ±1.87	110.83 ±3.48	110.01 ±3.15
RA (${}^{\circ }$)	112.67 ±2.17	110.09 ±1.91	108.56 ±2.92	108.06 ±4.00

**Table 7:** Kinetic variables in the hand-rim (mean ±SD).

	Paraplegic Group		Tetraplegic Group	
	WCh A	WCh B	WCh A	WCh B
$Ftot_{max}$ (N)	47.00 ±005.80	48.20 ±007.74	93.16 ±027.83	118.37 ±075.22
$Ftang_{max}$ (N)	36.12 ±006.89	38.98 ±004.92	43.18 ±007.30	48.61 ±009.92
$F_{eff}$	0.76 ±000.06	0.81 ±000.04	0.49 ±000.16	000.49 ±000.22
ERF (N/s)	908.87 ±311.47	816.95 ±157.74	2176.17 ±765.88	1958.45 ±665.80
$Mtot_{max}$ (Nm)	9.28 ±001.77	10.02 ±001.26	11.10 ±001.88	12.49 ±002.55
ERM (Nm/s)	212.48 ±068.56	180.83 ±036.92	227.37 ±073.49	222.50 ±076.83

**Table 8:** Forces and moments that show significance on the shoulder (median ±IQR).

	Paraplegic Group		Tetraplegic Group	
	WCh A	WCh B	WCh A	WCh B
Fxmin (N)	−6.80 ±2.28	−4.72 ±5.46	−27.48 ±14.95	−16.44 ±26.69
Fymax (N)	−2.20 ±7.01	−2.72 ±9.60	13.93 ±30.64	18.52 ±29.77
Fzmax (N)	4.17 ±1.03	4.84 ±1.86	9.78 ±03.20	8.25 ±02.39
Mxmax (Nm)	−1.25 ±0.20	−0.94 ±0.70	1.92 ±04.73	2.30 ±09.35
Mymin (Nm)	−0.18 ±0.11	−0.05 ±0.36	−1.46 ±00.33	−0.75 ±02.06
Mzmax (Nm)	3.80 ±2.55	5.31 ±2.51	10.08 ±05.86	13.60 ±06.83

**Table 9:** Kinematic variables on the shoulder: maximum, minimum and ROM values (mean ±SD) and the propulsion phase in which they happen: pushing (p) or recovery phase (r).

	Paraplegic Group		Tetraplegic Group	
	WCh A	WCh B	WCh A	WCh B
$\alpha_{sh,max}$ (${}^{\circ }$)	−27.18 ±06.49 (r)	−22.72 ±06.06 (p)	−28.45 ±03.04 (r)	−27.87 ±05.76 (r)
$\alpha_{sh,min}$ (${}^{\circ }$)	−39.09 ±07.76 (r)	−36.82 ±07.11 (r)	−45.83 ±08.35 (r)	−43.25 ±09.47 (r)
ROM $\alpha_{sh}$ (${}^{\circ }$)	11.91 ±01.86	14.10 ±08.17	17.38 ±09.13	15.38 ±09.20
$\gamma_{sh,max}$ (${}^{\circ }$)	39.26 ±23.89 (r)	20.27 ±30.83 (r)	28.30 ±35.60 (r)	46.09 ±21.69 (r)
$\gamma_{sh,min}$ (${}^{\circ }$)	−27.95 ±27.72 (r)	−56.26 ±16.87 (r)	−40.97 ±20.77 (r)	−20.08 ±29.63 (r)
ROM $\gamma_{sh}$ (${}^{\circ }$)	67.21 ±23.28	76.53 ±26.04	69.27 ±25.63	66.17 ±20.06
$\beta_{sh,max}$ (${}^{\circ }$)	28.14 ±23.81 (r)	47.32 ±17.15 (r)	47.90 ±14.00 (r)	37.29 ±21.02 (r)
$\beta_{sh,min}$ (${}^{\circ }$)	−32.75 ±13.02 (r)	−26.06 ±29.81 (r)	−17.15 ±40.31 (r)	−29.94 ±22.55 (r)
ROM $\beta_{sh}$ (${}^{\circ }$)	60.89 ±23.20	73.38 ±27.61	65.04 ±27.08	67.23 ±14.76

**Table 10:** Forces and moments that show significance on the elbow (median ±IQR).

	Paraplegic Group		Tetraplegic Group	
	WCh A	WCh B	WCh A	WCh B
Fymax (N)	23.07 ±11.72	24.45 ±7.00	34.81 ±16.48	51.03 ±13.88
Fzmax (N)	0.38 ±01.24	−0.37 ±2.37	9.28 ±08.55	5.31 ±13.63
Mymax (Nm)	0.18 ±00.11	0.05 ±0.36	1.46 ±00.33	0.75 ±02.06

**Table 11:** Kinematic variables on the elbow: maximum, minimum and ROM values (mean ±SD) and the propulsion phase in which they happen: pushing (p) or recovery phase (r).

	Paraplegic Group		Tetraplegic Group	
	WCh A	WCh B	WCh A	WCh B
$\gamma_{e,max}$ (${}^{\circ }$)	63.82 ±03.87 (p)	66.08 ±04.19 (p)	67.36 ±16.15 (p)	70.50 ±18.22 (p)
$\gamma_{e,min}$ (${}^{\circ }$)	42.07 ±11.29 (r)	41.38 ±07.94 (r)	25.15 ±04.15 (r)	34.73 ±15.53 (r)
ROM $\gamma_{e}$ (${}^{\circ }$)	21.76 ±13.69	24.70 ±06.68	42.21 ±16.55	35.77 ±18.06
$\beta_{e,max}$ (${}^{\circ }$)	124.22 ±15.90 (r)	134.32 ±06.69 (r)	127.17 ±22.60 (r)	119.48 ±37.23 (r)
$\beta_{e,min}$ (${}^{\circ }$)	98.44 ±18.10 (r)	103.07 ±10.89 (r)	66.37 ±17.92 (p)	67.82 ±05.87 (p)
ROM $\beta_{e}$ (${}^{\circ }$)	25.78 ±09.72	31.25 ±06.82	60.80 ±30.96	51.67 ±32.78

**Table 12:** Forces and moments that show significance on the wrist (median ±IQR).

	Paraplegic Group		Tetraplegic Group	
	WCh A	WCh B	WCh A	WCh B
Fymax (N)	28.31 ±5.77	28.62 ±10.83	54.81 ±17.22	61.33 ±7.15
Mxmin (Nm)	−0.25 ±0.07	−0.12 ±00.10	−0.54 ±00.21	−0.38 ±0.09

**Table 13:** Kinematic variables on the wrist: maximum, minimum and ROM values (mean ±SD) and the propulsion phase in which they happen: pushing (p) or recovery phase (r).

	Paraplegic Group		Tetraplegic Group	
	WCh A	WCh B	WCh A	WCh B
$\alpha_{wr,max}$ (${}^{\circ }$)	18.39 ±08.08 (r)	19.20 ±05.34 (p)	22.24 ±06.60 (p)	19.37 ±07.61 (p)
$\alpha_{wr,min}$ (${}^{\circ }$)	16.28 ±10.77 (r)	22.80 ±08.25 (r)	19.54 ±05.15 (r)	20.99 ±08.75 (r)
ROM $\alpha_{wr}$ (${}^{\circ }$)	6.23 ±06.51	9.32 ±05.29	6.74 ±05.55	10.33 ±08.26
$\gamma_{wr,max}$ (${}^{\circ }$)	10.85 ±09.67 (r)	15.72 ±04.81 (p)	11.43 ±10.52 (r)	5.19 ±06.85 (r)
$\gamma_{wr,min}$ (${}^{\circ }$)	19.50 ±04.25 (r)	16.43 ±05.86 (r)	25.03 ±20.97 (r)	32.16 ±24.09 (p)
ROM $\gamma_{wr}$ (${}^{\circ }$)	9.45 ±07.20	7.05 ±03.91	16.50 ±19.65	26.98 ±25.32
